# Burden of phenylketonuria in Latin American patients: a systematic review and meta-analysis of observational studies

**DOI:** 10.1186/s13023-022-02450-2

**Published:** 2022-07-30

**Authors:** A. L. S. Pessoa, A. M. Martins, E. M. Ribeiro, N. Specola, A. Chiesa, D. Vilela, E. Jurecki, D. Mesojedovas, I. V. D. Schwartz

**Affiliations:** 1 Albert Sabin Children’s Hospital / Ceara State University, Fortaleza, Ceará Brazil; 2 State University of Ceará (UECE), Fortaleza, Ceará Brazil; 3 Reference Center in Inborn Errors of Metabolism, Universidade Federal de São Paulo, São Paulo, Brazil; 4 Center Medical School, Christus University, Fortaleza, Ceará Brazil; 5 Hospital de Niños de La Plata, La Plata, Argentina; 6 Centro de Investigaciones Endocrinologicas “Dr Cesar Bergadá” CEDIE -CONICET- FEI: Division de Endocrinologia Hospital de Niños Ricardo Gutièrrez, Buenos Aires, Argentina; 7 BioMarin Farmacêutica, São Paulo, Brazil; 8 BioMarin Pharmaceutical Inc, Novato, CA USA; 9 Medical Genetics Service, HCPA, Rua Ramiro Barcelos, 2350, Porto Alegre, RS 90035-903 Brazil

**Keywords:** Neurological disease, Attention deficit hyperactivity disorder, Overweight, Phenylketonuria, LATAM

## Abstract

**Background:**

Phenylketonuria (PKU) is an inborn error of metabolism caused by a deficiency of the enzyme phenylalanine hydroxylase. If untreated, the complications of PKU lead to significant neucognitive and neuropsychiatric impairments, placing a burden on both the individual’s quality of life and on the healthcare system. We conducted a systematic literature review to characterize the impact of PKU on affected individuals and on healthcare resources in Latin American (LATAM) countries.

**Methods:**

Searches of the global medical literature as well as regional and local medical literature up to September 2021. Observational studies on patients with PKU from any LATAM country. Pairs of reviewers independently screened eligible articles, extracted data from included studies, and assessed their risk of bias.

**Results:**

79 unique studies (47 cross-sectional studies, 18 case series, 12 case reports, and two cohort studies) with a total of 4090 patients were eligible. Of these studies, 20 had data available evaluating early-diagnosed PKU patients for meta-analysis of burden outcomes. Intellectual disability in the pooled studies was 18% [95% Confidence Interval (CI) 0.04–0.38; I^2^ = 83.7%, *p* = 0.0133; two studies; n = 114]. Motor delay was 15% [95% CI 0.04–0.30; I^2^ = 74.5%, *p* = 0.0083; four studies; n = 132]. Speech deficit was 35% [95% CI 0.08–0.68; I^2^ = 93.9%, *p* < 0.0001; five studies; n = 162].

**Conclusions:**

There is currently evidence of high clinical burden in PKU patients in LATAM countries. Recognition that there are many unmet neuropsychological needs and socioeconomic challenges faced in the LATAM countries is the first step in planning cost-effective interventions.

**Supplementary Information:**

The online version contains supplementary material available at 10.1186/s13023-022-02450-2.

## Background

Phenylketonuria (PKU) is an inborn error of metabolism caused by a deficiency of the enzyme phenylalanine hydroxylase (PAH) which results in elevated levels of phenylalanine (Phe) and reduced levels of tyrosine [1]. The incidence within Latin American (LATAM) countries is estimated at 1 in 23,000 live births [2]. PKU presents a spectrum of severity, and there are several different classifications that have been proposed [2]. Several different classification schemes to determine clinical management have been proposed since PAH deficiency presents a spectrum of severity [1]. Individuals with classical PKU have a complete enzyme deficiency resulting in untreated blood Phe levels > 1200 μmol/L (an average normal Phe level is approximately 60 μmol/L), which is considered the severe form of this disorder [1].

The treatment for PKU is a lifelong dietary restriction of protein supplemented by a Phe-free amino acid fortified medical food [1], and ongoing monitoring of blood Phe levels to maintain a target range of 120–600 μmol/L for patients ≥ 12 years old, and up to 360 μmol/L for those < 12 years old [3]. If left untreated, the disease can manifest as significant intellectual impairment, neuropsychiatric disorders, and seizures [4] which place a burden on the individual’s quality of life, their families, and on the public and private healthcare systems [4]. Even patients diagnosed and treated at an early age face significant challenges related to adherence with the Phe-restricted diet.

Latin America comprises of 20 countries that represent a great diversity not only in terms of geography but also demographics, economies, languages, ethnicities, and health care systems [5]. In a recent review from Borrajo [6], newborn screening (NBS) programs were distinctively implemented in Latin America. While some programs date back from the 1980’s, other countries are still implementing regional NBS programs. Additionally, the number of diseases covered varies significantly across programs. Spefically concerning PKU, this genetic disorder is included in the NBS program for 14 countries (Cuba, Costa Rica, Chile, Uruguay, Argentina, Mexico, Brazil, Guatemala, Paraguay, Panama, Ecuador, Peru, Bolivia and Honduras) [6] with on average 92.3% of newborns in those countries screened for PKU. Regarding the availability of PKU treatment, half of the Latin America countries have fully subsidized medical foods by the government though the special low-protein foods are not available in most of the countries [7].

Evaluating the unmet needs and burden of PKU on affected individuals is important to determine the impact on the LATAM public and private healthcare system. Recognition that there are many challenges that the patient with PKU faces is the first step in planning for cost-effective intervention scenarios. We therefore conducted a systematic literature review and meta-analysis to better characterize the impact of PKU in LATAM countries on selected patient-important outcomes as well as at the economic (socioeconomic, healthcare utilization) level.

## Material and methods

Our review followed recommendations for systematic reviews and meta-analyses of observational studies in epidemiology (MOOSE) [8]. This systematic review has been registered in the PROSPERO (International Prospective Register of Systematic Reviews) database under the number CRD42020211417.

### Eligibility criteria

We included any epidemiological observational study (ie, cohort, case-control, nested case-control, cross-sectional studies, case series, case reports, surveys) on patients with PKU or phenylalanine hydroxylase deficiency (PAH), regardless of disease severity, including classical, moderate, and mild forms of this disorder, from any LATAM country regardless of whether they reported on any of the pre-defined patient-important outcomes and/or economic burden outcomes as defined below. We also included studies on caregivers of PKU patients.

Studies that only reported disease prevalence or incidence as well as non-human studies and subjective reports of clinical or observational studies such as letters, editorials and commentaries were excluded.

Pre-defined patient-important outcomes of interest included:

- Neurological, neurocognitive and neuropsychiatric impairments: intellectual disability, mental disorders, autism spectrum disorder, motor deficits, speech deficits and language delay, tremor, Attention Deficit Hyperactivity Disorder (ADHD) and hyperactivity, mood, depression, anxiety, phobias, irritability and/or aggressiveness, frustration, social isolation;
- Executive function deficit: working memory, sustained attention, inhibitory control, processing speed impairments, impairment in visuomotor coordination;
- Other comorbidities such as overweight, osteopenia, osteoporosis, skin problems, headaches, fatigue and sleeping disorder;
- Quality of life measured by non-validated and validated questionnaires, as defined by the included studies; and
- Patient adherence to clinical recommendations, including frequency of blood testing (ideally biweekly to monthly with targeted Phe concentrations of 120–360 μmol/L as recommended by the American College of Medical Genetics and Genomics (ACMG) guidelines [1] and 120–600 μmol/L for those ≥ 12 years of age by the European guidelines [9] and dietary management including a Phe-restricted diet supplemented by Phe-free amino acid fortified medical foods as well as the use of sapropterin dihydrochloride in patients who are responsive to this pharmacological treatment.
  Symptoms of being late-treated for the disease, such as seizures, microcephaly, generalized rash, and peculiar-smelling urine, were not investigated as patient-important outcomes for the purposes of this review.
  Pre-defined economic outcomes of interest included:
- Socioeconomic impact (eg, school / education level, work experience and productivity, marital status, personal independence, living situation, employment, social status);
- Impact of PKU on caregiver health-related quality of life; and
- Impact of PKU on the healthcare system (eg, direct and/or indirect costs, treatment costs, health care resource use, cost of comedications, hospitalizations).

### Data source and searches

Using Medical Subject Headings (MeSH) based on the terms “phenylketonuria” and “phenylalanine” (Additional file 1: Table 1) we performed the search in the global medical literature using Medical Literature Analysis and Retrieval System Online (MEDLINE, via PubMed, from 1946 to September 2021), Excerpta Medica Database (EMBASE, via Elsevier, from 1974 to September 2021), and Web of Science (to September 2021).

In the regional and local medical literature, both Spanish and English terms were used to search in Latin American and Caribbean Health Sciences Literature (LILACS, 1982 to September 2021), Scientific Electronic Library Online (SciELO, 1997 to September 2021), SciVerse Scopus via Elsevier (to September 2021), the Spanish Bibliographic Index of the Health Sciences (IBECS, 1983 to September 2021), National Bibliography in Health Sciences Argentina (BINACIS, to September 2021), Caribbean Health Sciences Literature (MedCarib, to September 2021), National Medical Sciences Information Center of Cuba (CUMED, to September 2021), Brazilian Bibliography of Dentistry (BBO, to September 2021), Health Information Locator (LIS, to September 2021), Regional Database of Health Technology Assessment Reports of the Americas (BRISA/RedTESA, to September 2021), Nursing Database (BDENF, to September 2021), Index Psychology (IndexPsi, to September 2021), and the WHO Institutional Repository for Information Sharing (WHO IRIS, to September 2021). The date the search was conducted was September 24, 2021 and no starting date restrictions, or language restrictions, were imposed. The search strategy was adapted for each database to achieve more specificity and sensitivity. Duplicate records across databases were removed.

We searched the gray literature including the Brazilian Digital Library of Theses and Dissertations (BDTD). In addition, reference lists of relevant primary studies were hand searched and experts in the field were contacted to obtain additional unpublished data where feasible.

### Selection of studies

**Fig. 1:**
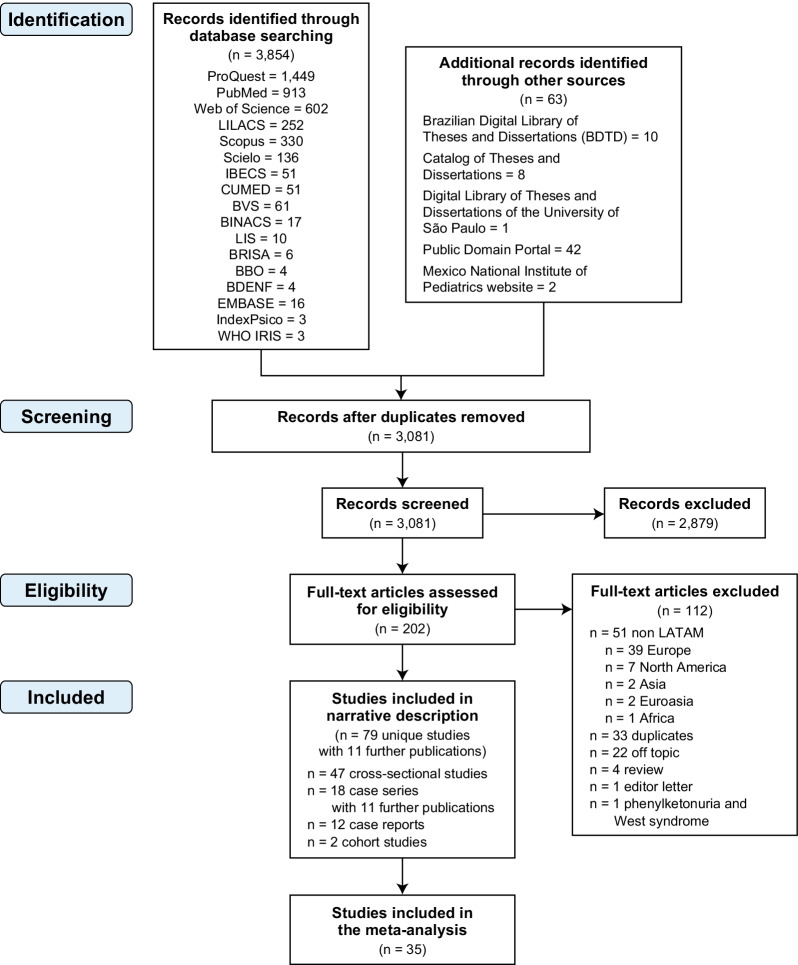
PRISMA flow diagram

Reviewers independently screened all titles and abstracts identified by the literature search using online software Covidence (https://www.covidence.org), obtained full-text articles of all potentially relevant studies, and evaluated them against the eligibility criteria. Reviewers resolved disagreement by discussion or, if necessary, with third party adjudication. We also considered studies reported only as abstracts and we attempted to contact study authors for additional information where needed. We recorded the selection process and documented via a PRISMA (Preferred Reporting Items for Systematic Reviews and Meta-Analyses) flow diagram (Fig. 1).

### Data synthesis and statistical analysis

We performed a systematic review of studies with pooled analysis of proportions [10, 11], using the method of Stuart-Ord (inverse double arcsine square root) with their respective 95% confidence intervals (CI). Only case series and cross-sectional studies were considered for quantitative analysis; case reports were excluded. To avoid bias related to the effect of delayed implementation of dietary management in late treated patients, only early-diagnosed patients (ie, diagnosed within first three months of life) were included. Studies that did not report whether the treatment was implemented at an early or late age were excluded from the meta-analysis as well as those that did not separate data for the early- or late-diagnosed patients.

Since we expected that there were both clinical and methodological differences among the included studies, a random-effects model [12] was used to perform the pooled analysis of proportions [10, 11]. The meta-analysis was performed with the StatsDirect software, version 2.8.0. (StatsDirect Ltd, Altrincham, Cheshire, UK).

## Results

### Study selection

Our initial searches identified 3917 citations (n = 3854 from electronic searches; n = 63 identified through the gray literature). After removing duplicates from different databases, 3081 potentially relevant articles were further assessed using title and abstract review. A total of 202 articles were identified for full text assessment. After screening the full texts, we included 79 studies with 11 further publications (ie, multiple publications of the same set of patients) (47 cross-sectional studies, 18 case series, 12 case reports, and two retrospective cohort studies) with a total of 4090 patients [7, 18-17-106]. The reasons for exclusion are listed in the PRISMA flow diagram (Fig. 1). When studies were presented in more than one publication, all applicable references were included.

Six of the included studies were published only as an abstract [13–19], ten studies as a thesis [20–29], and the majority (n = 57) were published as full-text in peer-reviewed journals [14, 28, 30–74]. Seven further studies [27, 29, 75–79] were published initially as a thesis followed by a full-text publication [34, 43, 80–83]. When information regarding risk of bias or other aspects related to study criteria were unavailable in the methods, we attempted to contact study authors for additional information.

### Study characteristics

Sixty-four of the 79 included studies reported at least one patient-important outcome at individual or population level, and they are displayed in Table 1 for study characteristics. Regarding study design, 18 were case series [22, 23, 33, 34, 40–42, 45, 54, 55, 58, 60, 70, 84–87], 47 cross-sectional studies [6, 7, 13–15, 17–21, 24, 26, 29, 30, 35–39, 43, 46, 47, 53, 56, 59, 61, 62, 67, 68, 71, 73, 75–77, 79–81, 88–91], 13 case reports [34, 44, 48, 51, 63, 64, 69, 74, 84, 87, 92–94], and two cohort studies [57, 95].

**Table 1:** All LATAM PKU studies evaluating at least one of the pre-specified patient-important or economic burden outcomes (N = 64)

Author, year	LATAM country	#of patients	Age, Mean^¥^ (SD), y	Female (%)	Phenotype (%)	Exclusion criteria	Individual and/or population outcomes	Type of burden outcomes	Specific outcomes	Early or late diagnosed**	Specify the type of treatment	Age (days) at start of treatment	Follow-up (months)	Reasons why the study was excluded from the analysis
Benítez et al. 2001 [32]	Uruguay	2	12	0.0	NR	NR	Individual and economic	Neurological, neurocognitive and neuropsychiatric impairments	Mental disorders—repetitive behaviors (rocking, flapping, etc.); motor deficits—march with aid; speech deficits—only emits a word	NR	NR	NR	NR	Not included as did not report whether the treatment was implemented at an early or late age
Bernal, 2017 [33]	Argentina	3	NR	100.0	NR (66.66) and classic (33.33)	NR	Individual and economic	Patient adherence to clinical recommendations	NR	Early (66.7%) and late (33.3%)	Phe-restricted diet	3.80**	NR	Not included as did not report data separately for early versus late treated
								Neurological, neurocognitive and neuropsychiatric impairments	Speech deficits; intellectual disability; aggressiveness; low frustration tolerance					
Cornejo et al. 1995 [42] ^$^	Chile	17	18.8	NR	NR	NR	Individual	Neurological, neurocognitive and neuropsychiatric impairments	Slight retardation; normal mental development	Early	Phe-restricted diet and education program	20.3**·	NR	Included in Fig. 3D
Cornejo et al. 2003 [41] ^$^	Chile	19	19.9^¢^	52.63	NR	NR	Individual	Neurological, neurocognitive and neuropsychiatric impairments	Low motor development	Early	Direct breast feeding, and a special formula without Phe	19.9**	6	Included in Fig. 3E
Cornejo et al. 2012 [40] ^$^	Chile	184	0 to 20₠	46.73	Classic and moderate (NR)	NR	Individual	Overweight and obesity	–	Early	Phe-restricted diet and education program	18	NR	Included in Fig. 4A and B
								Neurological, neurocognitive and neuropsychiatric impairments	Average total IQ in preschoolers, schoolers and teenagers					
Diament & Lefevre, 1967 [45]	Brazil	6	4.36	66.66	NR	NR	Individual	Neurological, neurocognitive and neuropsychiatric impairments	Irritability; language delay; mental retardation; hyperactive patient	Late	Phe-restricted diet	NR	NR	Not included as population was not early diagnosed/treated
Figueira, 2018 [22] ^$^	Brazil	78	9.2	59.0	Classic (100.0)	Patients with less than 4 consultations in medical records and whose medical records are not filled out	Individual	Patient adherence to clinical recommendations	Non-adherence to Phe-restricted diet	Early (56.4%) and late (43.6)	Phe-restricted diet	NR	NR	Included in Figs. 3C, E, F, and 4E Not included as did not report data separately for early versus late treated
								Neurological, neurocognitive and neuropsychiatric impairments	Learning disability; neuropsychomotor development delay; neuromotor restriction; aggressivenes; autistic behaviour; speech deficits					
Gelvez et al. 2016 [54]	Colombia	4	13	75.0	Classic (100.0)	NR	Individual and economic	Neurological, neurocognitive and neuropsychiatric impairments	Speech deficits; neuropsychomotor development delay; aggressiveness; anxiety; attention deficit symptoms; executive function deficit	Late	Phe-restricted diet and education program	13**	NR	Not included as population was not early diagnosed/treated
								Skin problems	Hypopigmentation					
								Socioeconomic impact	Poor school performance					
Jiménez-Péres et al. 2015 [55] ^$^	Mexico	6	7	33.33	Classic (100.0)	NR	Individual	Neurological, neurocognitive and neuropsychiatric impairments	Global neurodevelopment impairment	Late	NR	690	NR	Included in Fig. 4C Not included as population was not early diagnosed/treated
								Skin problems	Eczema skin; lightening of the skin					
Lamônica et al. 2012 [58] ^$^	Brazil	10	NR	40.0	NR	Chronic disease	Individual	Executive function deficit	Receptive auditory, expressive auditory and visual functions	Early	Phe-restricted diet and mixed formula	Before 30 days of life	NR	Included in Figs. 3A, C; 4D, and 5
Mahfoud et al. 2008 [60] ^$^	Venezuela	5	NR	40.0	Classic (60.0) and mild (40.0)	NR	Individual	Neurological, neurocognitive and neuropsychiatric impairments	Global development impairment; isolation; hyperactivity; speech deficits	Early	Phe-restricted diet and special formula	480	NR	Included in Fig. 3A, C, F, and 4D
								Executive function deficit	Irritability; sleeping disorder					
Martins, 2007 [23]	Brazil	15	9 to 29₠	66.7	NR	NR	Individual	Overweight and obesity	–	NR	Phe-restricted diet	NR	6	Not included as did not report whether the treatment was implemented at an early or late age
								Osteopenia	Osteopenia					
Queiroz & Pondé, 2015 [84]	Brazil	8	NR	37.5	NR	NR	Individual	Neurological, neurocognitive and neuropsychiatric impairments	Hyperactivity; attention deficit; aggressiveness; intellectual disability; autism	Early (50%) Late (50%)	Phe-restricted diet	3,698**	NR	Not included as did not report data separately for early versus late treated
Sánchez-Peña et al. 2008 [85]	Mexico	3	5.6	100.0	Classic (66.6) and moderate (33.3)	NR	Individual	Neurological, neurocognitive and neuropsychiatric impairments	Personal-social development delay; adaptive-motor delay; language delay; hyperactivity; aggressiveness; irritability; autistic behaviour	Early (66.7%) Late (33.3%)	Phe-restricted diet and special formula	2,044**	At least 3	Not included as did not report data separately for early versus late treated
Silva et al. 2016 [70]	Brazil	36	NR	52.77	NR	NR	Individual	Patient adherence to clinical recommendations	Noncompliance to treatment	Early (80.55%)	Phe-restricted diet	NR	NR	Not included as quantitative data on outcome of interest not provided in paper
Steiner et al. 2007 [86]	Brazil	3	17.33	33.33	NR	NR	Individual and economic	Neurological, neurocognitive and neuropsychiatric impairments	Body shaking; autism spectrum disorder; no verbal language; aggressivenes; hyperactivity	Early	Phe-restricted diet	NR	NR	Not included as quantitative data on outcome of interest not provided in paper
								Socioeconomic impact	School for autistic children					
								Impact of PKU on caregiver health-related quality of life	Did not acquire toilet training					
Tanaka et al. 2018 [72]	Brazil	18	10	39.0	NR	Patients who did not adhere to the dietary treatment (evaluated by the food anamnesis) associated with no intake of elemental formula free of Phe in the recommended amount and those who were receiving a drug supplement of calcium	Individual	Overweight or obese	–	NR	Phe-restricted diet and special formula	NR	34^¢^	Not included as did not report whether the treatment was implemented at an early or late age
Valle et al. 2019 [87]	Argentina	133	2 months to adulthood	NR	Moderate (30.8), mild (67.6), and HPA (33.08)	NR	Individual	Neurological, neurocognitive and neuropsychiatric impairments Others	Neurocognitive evaluation Successful pregnancies Patient adherence to clinical recommendations	Early (24.06%) and late (3.75%)	Phe-restricted diet + protein substitute (54.13%); Phe-restricted diet + glycomacropeptides (1.50%); and diet counselling (31.57%); BH4 (9.77%)	NR	Until age five and monthly thereafter	Not included as did not report data separately for early versus late treated
Andere et al. 1988£ [96] ^$^	Brazil	35	4* to 11₠	48.57	Classic (100.0)	NR	Individual	Skin problems	Keratosis pilaris, ammonia dermatitis, dry skin, reticular livedo and dermographism; during the dietary treatment darkening of skin, hair and eyes; lightening of the skin and hair	NR	Phe-restricted diet	NR	NA	Included in Fig. 4C
Beckhauser et al. 2020 [31] ^$^	Brazil	34	12	47.0	NR	Patient who started treatment after 60 days of age, who failed to maintain Phe levels below 6 mg/dL or who failed to adhere to regular medical follow-ups	Individual	Neurological, neurocognitive and neuropsychiatric impairments	ADHD	Early	Regularly treated since birth according to the “Brazilian Phenylketonuria Clinical and Therapeutic Guidelines”, consisting of a diet and protein formula diet, with Phe restrictions	Before 60 days of life (treated since birth)	NA	Included in Fig. 3A
Brandalize, 2004 [75, 88]	Brazil	32	0 to 6₠	56.3	NR (84.40) and moderate (16)	Children who started early treatment in the pioneering program of the Association of Parents and Friends of the Exceptional of São Paulo (APAE-SP), late diagnoses, and early diagnosis due to age above the rest of the group (11, 13 and 14 years)	Individual	Neurological, neurocognitive and neuropsychiatric impairments	Low development of gross motor function; mean gross motor function of PKU and HPAP children; for PKU and HPAP, low development of gross motor function	Early	Phe-restricted diet	For PKU, 8 days to 30 days after born (n = 23 patients) and 31 days to 60 days after born (n = 4 patients); for HPAP, 2 months to 1 year after born (n = 5 patients)	NA	Not included as quantitative data on outcome of interest not provided in paper
Pardo-Campos et al. [35–37]^£^	Argentina	30	8 to 11₠	10.4	NR	NR	Individual and economic	Executive function deficit Impact of PKU on caregiver health-related quality of life	Coping strategies (facing conflicts, relationship with impulsivity); cognitive profile; organization; IQ; memory, visuospatial skills, reaction times, processing speed or in language Parenting styles perceived by the children	Early	NR	NR	NA	Not included as quantitative data on outcome of interest not provided in paper
Chiesa et al. 2012 [38]	Argentina	NR	NR	NR	NR	NR	Individual	Neurological, neurocognitive and neuropsychiatric impairments	NR	NR	Phe-restricted diet, and free animal food	NR	NA	Not included as quantitative data on outcome of interest not provided in paper
Camatta, 2020 [76, 80] ^$^	Brazil	94	14	53.0	NR	Tetrahydrobiopterin (BH4) deficiency, use of pacemaker, pregnancy, growth-related disorder, and abandonment of treatment over the two previous years	Individual	Overweight and obese	–	Early	Phe-restricted diet and special formula	Up to 30 days of life	NA	Included in Fig. 4A
Castro et al. 2012 [77, 81]	Brazil	63	6 to 12₠	52.4	Classic (82.5) and mild (17.5)	Not having a free and informed consent form; child's disagreement; and lack of information on Phe dosages of the transferred patients	Individual	Neurological, neurocognitive and neuropsychiatric impairments	Intellectually disabled from total IQ	Early	Phe-restricted diet	Up to 90 days after born	NA	Not included as quantitative data on outcome of interest not provided in paper
Cerqueira, 2004 [20]	Brazil	101	34.23^§^	84.2	NA	NR	NR	NR	NR	NR	NR	NR	NA	Not included as did not report whether the treatment was implemented at an early or late age
Colombo et al. 1988 [39] ^$^	Chile	44	3.11	NR	NR	NR	Individual	Neurological, neurocognitive and neuropsychiatric impairments	Language delay; isolated psychomotor developmental delay; mental retardation; hyperactivity; irritability; psychomotor or mental retardation; psychometric evaluation; MRI	Late	Phe-restricted diet	3 years 11 months	NA	Included in Fig. 3A, C, and D Not included as population was not early diagnosed/treated
Da Silva et al. 2020 [43]	Brazil	31	17.4	51.6	Classic (30.8) and mild (69.2)	To be in an irregular clinical follow-up in the last 12 months; (2) to have a clinical diagnosis of intellectual disability or diagnosis of other associated genetic, psychiatric, or neurological diseases which compromise the assessment of ADHD	Individual	Neurological, neurocognitive and neuropsychiatric impairments	ADHD	Late	Phe-restricted diet and special formula	26**	NA	Not included as population was not early diagnosed/treated
Dutra et al. 2013£ [21]	Brazil	21	9.52	42.9	Classic (4.76) and mild (57.14) and HPAP (38.1)	Children and adolescents whose parents or legal guardians have not signed the ICF; with confirmed diagnosis of neurological and / or psychiatric illness or other syndromes that cause delays in cognitive development	Individual	Neurological, neurocognitive and neuropsychiatric impairments	Neuropsychological assessment; executive function of IQ; verbal of IQ; verbal comprehension index; perceptual organization index; distraction resistance index; Stroop	Early	NR	Up to 90 days after born	NA	Not included as quantitative data on outcome of interest not provided in paper
								Executive function deficit	Processing speed index; RAVLT total score; Late RAVLT; RAVLT recognition; RVDLT total score; Late RVDLT; RVDLT recognition; time for test (seconds) for TMT A and B; number of errors for TMT A and B					
Gejão et al. 2009 [53] ^$^	Brazil	25	1 to 10₠	NR	NR	NR	Individual	Neurological, neurocognitive and neuropsychiatric impairments	Alterations in fine motor adaptative, gross motor, language, and personal-social behaviour; motor alterations; language alterations; cognitive alterations; self-care alterations; socialization alterations; alterations in expressive auditory, receptive auditory, and visual auditory; LDES; alterations in PPVT, and Total Score ABFW Child Language Test-phonology; alterations in Visual reception, auditory association, visual association, auditory memory, visual memory, auditory closure, grammatical closure, visual closure, verbal expression, manual expression, sounds combination; difficulty in attention time maintenance; hyperactivity	Early	According to national guidelines	Up to 30 days of life	NA	Included in Figs. 3A, E, and F
Kanufre et al. 2015 [56]	Brazil	58	9.15	48.27	NR	NR	Individual	Overweight	Overweight	NR	Phe-restricted diet	NR	NA	Not included as did not report whether the treatment was implemented at an early or late age
Keselman 2005 et al. [17]^£^	Argentina	11	8.7 to 13₠	18.28	Classic (100.0)	NR	Individual	Osteopenia	Bone mineralization and lumbar spine	Early	Phe-restricted diet	NR	NA	Not included as quantitative data on outcome of interest not provided in paper
Lamônica et al. 2015 [59] ^$^	Brazil	17	10.2	36.0	Classic (100.0)	NR	Individual and economic	Neurological, neurocognitive and neuropsychiatric impairments	ADHD; IQ; low Reading School Performance Test—Number of patients classified as Inferior; low Writing School Performance Test—Number of patients classified as Inferior; irritability	Early	Phe-restricted diet	76.47% before 30 days of life and 23.53% after 30 days of life	NA	Included in Figs. 3A, C; 4D, and 5
								Executive function deficit	Peabody Picture Vocabulary Test—Number of patients classified as Low					
								Sleeping disorders	Sleeping disorder					
								Socioeconomic impact	Poor school performance					
Malloy-Diniz et al. 2004 [61] ^£^	Brazil	21	274^¢^	61.9	NR	Average phe level below 120 μmol/l	Individual	Neurological, neurocognitive and neuropsychiatric impairments	Bayley Scale of Infant Development	Early	Phe-restricted diet	27.5**	NA	Not included as quantitative data on outcome of interest not provided in paper
								Executive function deficit	A not B task Piaget					
Mancini et al. 2010 [62] ^£$^	Brazil	33	7.67	52.38	NR	NR	Individual	Patient adherence to clinical recommendations	Serum PKU	Early	Phe-restricted diet	Up to 21 days of life	NA	Included in Fig. 4E
Martins et al. 2020 [30]	Brazil	228	Newborn, 90%; between 1 and 5 years old, 8%; and over 10 years old (2%)	21.49	NR	NR	Individual and economic	Neurological, neurocognitive and neuropsychiatric impairments Socioeconomic impact; the impact of PKU on the daily lives of patients and caregivers; the main difficulties faced by PKU patients and their caregivers; cognitive and emotional symptoms	Irritability, anxiety, and lack of concentration Financial impact related to the PKU management; stop working to care for the PKU patient; need to hire a caregiver to assist the PKU patient; absence of neuropsychological care; did not receive the support of a day-to-day psychologist; limitation on social activities; impact on professional life; and effect on self-esteem	Early (89.92%) and late (10.08%)	Phe-restricted diet and supplements	NR	NA	Not included as did not report data separately for early versus late treated
Mendes, 2006 [19]	Brazil	17	NR	70.58	NR	NR	Individual	Osteopenia	Osteopenia	NR	Phe-restricted diet	NR	NA	Not included as did not report whether the treatment was implemented at an early or late age
Morão, 2017 71]	Brazil	20	NR	NR	NR	NR	Individual	Neurological, neurocognitive and neuropsychiatric impairments	Verbal fluency test; Children Behavior Checklist 6/18; Wechsler Intelligence Scale for Children (WISC-IV); Word and Pseudoword Reading Competency Test; Snap automatic naming test; SNAP—IV (attention deficits)	NR	NR	NR	NA	Not included as did not report whether the treatment was implemented at an early or late age
								Executive function deficit	Children Gambling Task; delay of gratification adapted; Rey complex figure; five digit test; RAVLT					
Nalin et al. 2010 [66]	Brazil	45	11	49.0	NR (18.0) and classic (53.0) and mild (29.0)	NR	Individual	Patient adherence to clinical recommendations	Patient adherence to treatment	Early	Phe-restricted diet and special formula	90	NA	Not included as quantitative data on outcome of interest not provided in paper
Viera Neto et al. 2018 [67] ^£ $^	Brazil	51	6 to 17₠	43.13	NR (2.0) and classic (64.7) and mild/moderate (33.3)	NR	Individual	Neurological, neurocognitive and neuropsychiatric impairments	Intellectual capacity classified as below average or intellectual defective	Early	Phe-restricted diet and special formula	48	NA	Included in Figs. 3B and 4E
								QoL	Total score; physical health; emotional functioning; social functioning; school functioning; psychosocial health					
								Patient adherence to clinical recommendations	Adequate serum PKU levels					
Paneque et al. 2013 [68]	Cuba	12	NR	NR	NR	NR	Individual and economic	Neurological, neurocognitive and neuropsychiatric impairments	Intelligence test, group attention test, and psychometric test (Weil's non-verbal intelligence test)	NR	Phe-restricted diet	NR	NA	Not included as did not report whether the treatment was implemented at an early or late age
								Overweight	Overweight according to the growth and development tables of the Cuban population (weight for height, height for age and weight for age)					
								Skin problems	Skin alterations					
								Osteopenia	Bone alterations					
								Socioeconomic impact	Current worker					
Peredo et al. 2010 [89]	Chile	20	13.4	100.0	Classic (100.0)	NR	Individual	Neurological, neurocognitive and neuropsychiatric impairments	IQ	Early	Special milk-based formula	17.9**	NA	Not included as quantitative data on outcome of interest not provided in paper
								Overweight	Overweight					
Pérsico et al. 2019 [97]	Brazil	15	16	53.33	Classic (53.3) and mild (46.7)	Presence of associated comorbidities and/or use of medications unrelated to specific diet therapy with the possibility of interfering with bone metabolism	Individual	Overweight	Overweight	NR	Phe-restricted diet, special formula, and supplement	NR	NA	Not included as did not report whether the treatment was implemented at an early or late age
Poloni et al. 2021 [7]	Brazil, Argentina, Colombia, Venezuela, Costa Rica, Chile, Mexico, Paraguay, Peru, Dominican Republic, Panama, Uruguay, and Cuba	NR	NR	NR	NR	NR	Individual and economic	Poor adherence Low purchasing power, limited/insufficient availability of low-protein foods, lack of technical resources to manage the diet, and did not have low-protein foods; no alternative treatments available	–	NR	Phe-restricted diet, unflavored powdered amino acid substitutes	NR	NA	Not included as did not report whether the treatment was implemented at an early or late age
Sena, 2018 [26]	Brazil	31	6.5	48.4	NR	Patients who were not accompanied by legal guardians advised at the time of collection, individuals hospitalized in any hospital units and patients who were diagnosed with chronic non-communicable diseases such as hypertension, diabetes and cancer	Individual	Overweight	Overweight	NR	Phe-restricted diet	NR	NA	Not included as did not report whether the treatment was implemented at an early or late age
Silva, 2010£ [98] ^£ $^	Brazil	10	5.18	50.0	Classic (100.0)	NR	Individual	Neurological, neurocognitive and neuropsychiatric impairments	Hyperactivity; attention deficit; below average for personal-social area; below average for adaptative, language, gross motor, and fine motor; vocabulary classified as below average; mild to moderate speech disorder; deficit for personal-social area, fine motor-adaptative area, language area, and gross motor area	Early	Phe-restricted diet	5.18**	NA	Included in Fig. 3A, E, and F
Silva, 2016 [91]	Brazil	24	15.8	50.0	Classic (50.0) and mild (50.0)	There was no exclusion criterion for samples	Individual	Neurological, neurocognitive and neuropsychiatric	Neuropsychomotor impairment; behavioral alterations	Early	Phe-restricted diet and special formula	92.29**	NA	Not included as quantitative data on outcome of interest not provided in paper
Silva, 2018 [78]^$^	Brazil	31	17.4	51.6	Classic (51.6) and mild (48.4)	Have a clinical diagnosis of mild, moderate, severe or profound intellectual disability; have a diagnosis of other associated genetic diseases, depression, bipolar mood disorder or epileptic encephalopathy	Individual	Neurological, neurocognitive and neuropsychiatric	ADHD Osteopenia	Early	Phe-restricted diet and special formula	26	NA	Included in Fig. 3A
								Osteopenia						
Silveira et al. 2021 [71]^$^	Brazil	101	14.0	45.5	Classic (56.4) and mild (43.6)	Patients with late diagnosis and patients diagnosed with tetrahydrobiopterin (BH4) deficiency	Individual	Overweight and obesity	–	Early	NR	NR	NA	Included in Fig. 4A, and B
Teruya, 2019 [79, 82]	Brazil	23	18	39.0	Classic (47.8) and mild (52.2)	NA	Not included as quantitative data on outcome of interest not provided in paper	Patient adherence to clinical recommendations	Poor current adherence to Phe-restricted diet; poor current median adherence to Phe-restricted diet	Early (65.2%)	Phe-restricted diet and special formula	Up to 90 days after born		
Tonon et al. 2019 [73]^£^	Brazil	25	19.3	52.0	Classic (52.0) and mild (48.0)	NR	Individual	Overweight or obese	–	Early	Phe-restricted diet and special formula	52.8**	NA	Not included as quantitative data on outcome of interest not provided in paper
Vieira, 2010 [29]^$^	Brazil	56	12	55.35	NR (12.5), classic (58.9) and mild (28.6)	NR	Individual and economic	Neurological, neurocognitive and neuropsychiatric	Mental retardation; learning disability; hyperactivity; aggressiveness; attention deficit	Early	Phe-restricted diet and special formula	60	NA	Included in Figs. 3A, C, D, 4E, and 5
								Patient adherence to clinical recommendations	Nonadherent to treatment					
								Socioeconomic impact	Special Education					
Blanco et al. 2012 [34]	Argentina	1	34	100.0	Mild (100.0)	NR	Individual	Executive function deficit	Mental retardation mild-moderate	Late	Phe-restricted diet	34 years	3	Not included due to design
De Lucca et al. 2017 [44]	Ecuador	1	15	100.0	NR	NR	Individual	Neurological, neurocognitive and neuropsychiatric impairments	Autism; psychomotor retardation	Early	Phe-restricted diet and special formula	3 years and 11 months	NR	Not included due to design
								Executive function deficit	Delayed severe mental					
								Skin problems	Musty smell; hair hypopigmentation					
Escaf, 2003 [48]	Colombia	1	NR	NR	HPAP (100.0)	NR	Individual	Neurological, neurocognitive and neuropsychiatric impairments	Irritability; sporadic seizures	NR	Phe-restricted diet	NR	NR	Not included due to design
								Skin problems	Eczema					
								Others	Vomiting					
Figueiró-Filho et al. 2004 [51]	Brazil	1	22	100.0	NR	NR	Individual	Others	Maternal PKU	Late	Phe-restricted diet and supplementation with protein hydrolyzate	22	NR	Not included due to design
Mariño & Zarzalejo, 2000 [63]	Venezuela	1	0.1	0.0	HPA (100.0)	NR	Individual	Neurological, neurocognitive and neuropsychiatric impairments	Motor deficits; mental development	Early	Breastfeeding and special formula	28	9	Not included due to design
								Skin problems	Erythema					
								Patient adherence to clinical recommendations	Adherence to Phe-restricted diet					
Menezes et al. 2019 [64]	Brazil	1	82^¢^	100.0	Classic (100.0)	NR	Individual	Neurological, neurocognitive and neuropsychiatric impairments	Psychomotor retardation; language impairment	Early	Phe-restricted diet and special formula associated with the milk formula	40	NR	Not included due to design
								Executive function deficit	Adynamia					
								Skin problems	Pale skin; hair loss					
								Patient adherence to clinical recommendations	Poor acceptance of the diet					
								Others	Weight-height deficit; vomiting					
Patricio & Maritza, 2018 [92]	Ecuador	1	29^Φ^	0.0	Classic (100.0)	NR	Individual	Neurological, neurocognitive and neuropsychiatric impairments	Seizures; neurological complications	Early	Phe-restricted diet and special formula	30	6	Not included due to design
								Executive function deficit	Axial hypotonia					
								Others	Persistent respiratory acidosis; abdominal distention					
Pereda-Torales et al. 2008 [93]	Mexico	1	0.2	0.0	Classic (100.0)	NR	Individual	Neurological, neurocognitive and neuropsychiatric impairments	Neuropsychomotor development	Early	Phe-restricted diet and special formula	60	12	Not included due to design
								Patient adherence to clinical recommendations	Adherence to Phe-restricted diet					
Rasner et al. 2014 [90]	Uruguay	1	10*	0.0	Classic (100.0)	NR	Individual	Neurological, neurocognitive and neuropsychiatric impairments	Losing the cephalic support; irritability; myoclonia of all four members; hypotonia	Early	NR	10 months	NR	Not included due to design
								Executive function deficit	Regression of the tracking with the look; regression picking up objects					
Santos & Haack, 2013 [94]	Brazil	1	5	100.0	Classic (100.0)	NR	Individual	Neurological, neurocognitive and neuropsychiatric impairments	Seizures	Early	Phe-restricted diet and special formula	20	60	Not included due to design
								Others	Gastroesophageal reflux; bronchitis; vomiting					
Schmidt et al. 2016 [69]	Brazil	1	13	0.0	NR	NR	Individual	Neurological, neurocognitive and neuropsychiatric impairments	Hypoactivity	Early	Phe-restricted diet	NR	NR	Not included due to design
								Others	Megaloblastic anemia					
Urbanes et al. 2006 [74]	Colombia	1	8	0.0	Classic (100.0)	NR	Individual	Neurological, neurocognitive and neuropsychiatric impairments	Restless; aggressive; inconsistent language; neuromotor restriction; intellectual deterioration; poor socialization	Late	NA	NA	NR	Not included due to design

*ADHD* Attention Deficit Hyperactivity Disorder, *HPA* hyperphenylalaninemia, *HPAP* hyperphenylalaninemia persistent, *IQ* intelligence quotient, *LATAM* Latin America, *LDES* Total Score Language Development Evaluation Scale, *NR* not reported, *NA* not applicable, *Phe* phenylalanine, *PKU* phenylketonuria, *PPVT* alterations in Total Score Peabody Picture Vocabulary Test, *RAVLT* Rey auditive verbal learning test, *RVDLT* Rey visual design learning test, *SD* standard deviation, *TMT* (partes A e B) Trail making test (partes A e B), *USP* Universidade de São Paulo, *UFMG* Universidade Federal de Minas Gerais, *y* years

***We considered that late diagnosed refers to children diagnosed between the ages of 3 months to 7 years (≥ 3 months to < 7 years); untreated PKU refers to patients untreated by 7 years of age and over

¢Days

*Months

**Mean

·Only in two was it done after 30 days

#Number

₠Range

£Comparative cross-sectional studies

ΦWeeks of gestational age

§Caregivers’ age

¥For the case reports, age is expressed as absolute number

$Included in the analysis

Forty of the included studies were conducted in Brazil [14, 19–24, 26, 27, 29, 31, 43, 45, 51, 56, 58, 59, 62, 64, 67, 69–73, 75–78, 80, 81, 83, 84, 86, 88, 91, 94, 97, 98], seven in Argentina [15, 17, 18, 33–38, 46, 47], five in Chile [39–42, 89], three each in Colombia [48, 54, 74] and Mexico [55, 85, 93], two each in Ecuador [44, 92], Venezuela [60, 63] and Uruguay [32, 90], and one in Cuba [68]. One study [87] was a multicenter conducted in Ecuador, Bolivia, and Paraguay. The case series and cross-sectional studies sample size ranged from two [32] to 420 patients [46, 47]. Patients’ ages ranged from a mean of 3.11 [20] to 19.3 [73] years old (Table 1).

The type of patient-important outcomes most frequently reported among the cross-sectional and case series studies were neurological, neurocognitive and neuropsychiatric impairments (n = 33 studies, 50.8%) [21, 22, 24, 27, 30–33, 38–40, 43, 45, 53–55, 59–61, 67, 68, 77, 78, 81, 83–89, 91, 98], followed by overweight (n = 11, 16.9%) [23, 26, 40, 56, 68, 71–73, 76, 80, 89, 97], patient adherence to clinical recommendations (n = 10, 15.4%) [7, 22, 33, 62, 66, 67, 70, 79, 82, 83, 87], executive function deficit (n = 6, 92.3%) [21, 24, 58–61], socioeconomic impact (n = 7, 10.8%) [7, 29, 30, 54, 59, 68, 83, 86], skin problems (n = 4, 6.2%) [54, 55, 68, 96], osteopenia (n = 5, 7.7%) [17–19, 23, 27, 68, 78], followed by impact of PKU on caregiver health-related quality of life, quality of life and sleeping disorders (n = 2, 3.1%) [30, 59, 67, 86]. The majority of the cross-sectional, case series, and case report studies (83.1%, n = 64) reported only on patient-important outcomes at an individual level (Table 1).

Among the 12 case report studies, the majority (83.3%, n = 10) assessed neurological, neurocognitive and neuropsychiatric impairments [44, 48, 63, 64, 74, 90, 92–94], executive function deficit [34, 44, 64, 90, 92], skin problems [43, 48, 63, 64], and patient adherence to clinical recommendations [23, 25, 61]. Six case report studies [48, 51, 64, 69, 92, 94] evaluated other outcomes such as vomiting [48, 64, 94], weight-height deficit [64], abdominal distention [68], persistent respiratory acidosis [68], gastroesophageal reflux [94], bronchitis [94], megaloblastic anemia [69], and maternal phenylketonuria [51] (Table 1).

Additional file 2: Table 2 describes the 15 studies evaluating other patient-important or economic burden outcomes than those pre-specified for this review; all of these, except for three studies [15, 46, 47, 49], reported that patients received a Phe-restricted diet and/or a Phe-free amino acid fortified medical food. Six studies out of 15 did not report whether patients were receiving treatment [15, 25, 46, 47, 52, 65].

Additional file 3: Table 3 provides additional details around the specific pre-specified patient-important or economic burden outcomes reported among the 12 case report studies. Five out of the 12 studies reported psychosocial outcomes (ie, severe mental retardation and autism, irritability, aggressiveness and intellectual deterioration) [44, 48, 64, 74, 90]; four on physical outcomes (ie, psychomotor retardation) [44, 64, 74, 90]; one study reported on other outcomes (ie, maternal phenylketonuria) [51]; and two studies reported socioeconomic results (ie, delay in school performance, poor socialization, and withdrawal from formal schooling) [64, 74].

### Risk of bias assesment

**Fig. 2:**
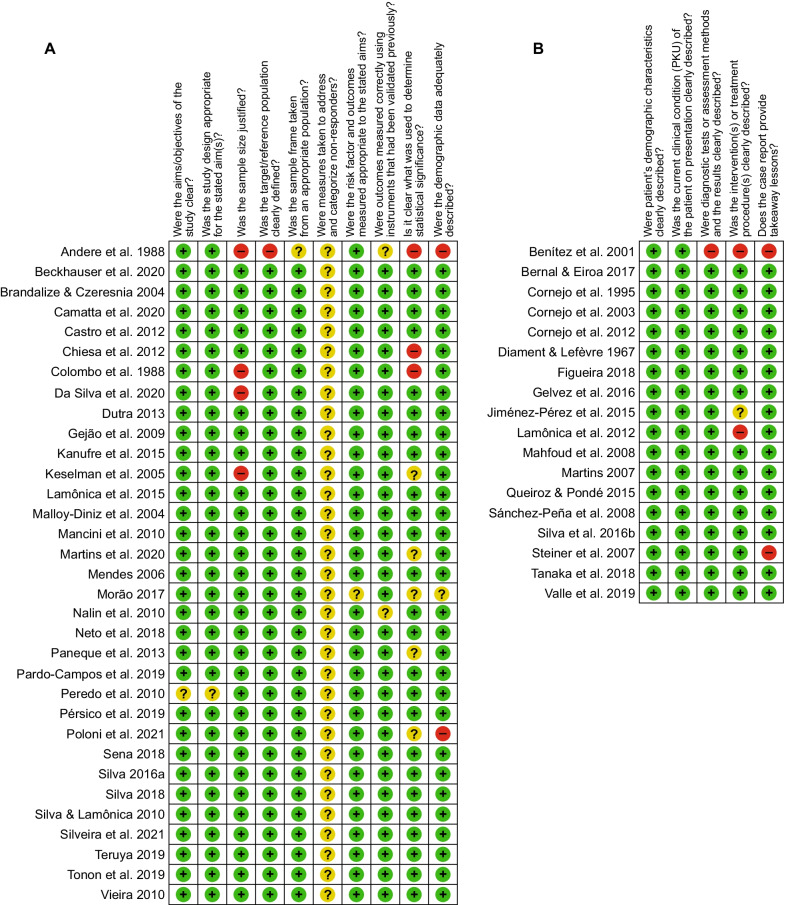
Risk of bias assessment. (**A**) cross-sectional studies. (**B**) case series studies

Figure 2 and Additional files 4 and 5: Tables 4 and 5 describe the risk of bias assessment. Overall, the included studies presented a low risk of bias in the majority of the domains. In the cross-sectional studies (Fig. 2, panel A), at least one of the following domains of sample size, statistical significance, statistics methods, or demographic data were rated as “high risk of bias” in five studies (12.8%) [7, 38, 39, 43, 96]. In the case series studies (Fig. 2, panel B), three domains (ie, clear description of both patient’s history and post-intervention clinical condition, and description of a takeaway lesson) were rated as “high risk of bias” in three studies (25.0%) [32, 58, 86].

### Meta-analysis results

The results were pooled from case series and cross-sectional studies that reported data only on early-diagnosed patients to avoid bias related to the effects of delayed implementation of dietary management in late-diagnosed patients. Studies that did not provide quantitative data on outcome of interest in papers were also excluded from analysis as well as studies that did not report whether the treatment was implemented at an early or late age. Therefore, out of 67 included studies [14, 16, 19–29, 31–34, 39–45, 48–50, 52–57, 59–70, 72–86, 88–101], 20 studies [22, 29, 31, 39–42, 52, 55, 59, 60, 62, 66, 67, 76, 77, 77, 78, 80, 83, 96, 98] qualified for the quantitative analysis described below. None of the included studies evaluating early-diagnosed PKU patients reported symptoms including headache and fatigue, quality of life, or the impact of PKU on the healthcare system.

#### Neurological, neurocognitive, and neuropsychiatric impairments

##### Attention deficit hyperactivity disorder (ADHD) and hyperactivity

**Fig. 3:**
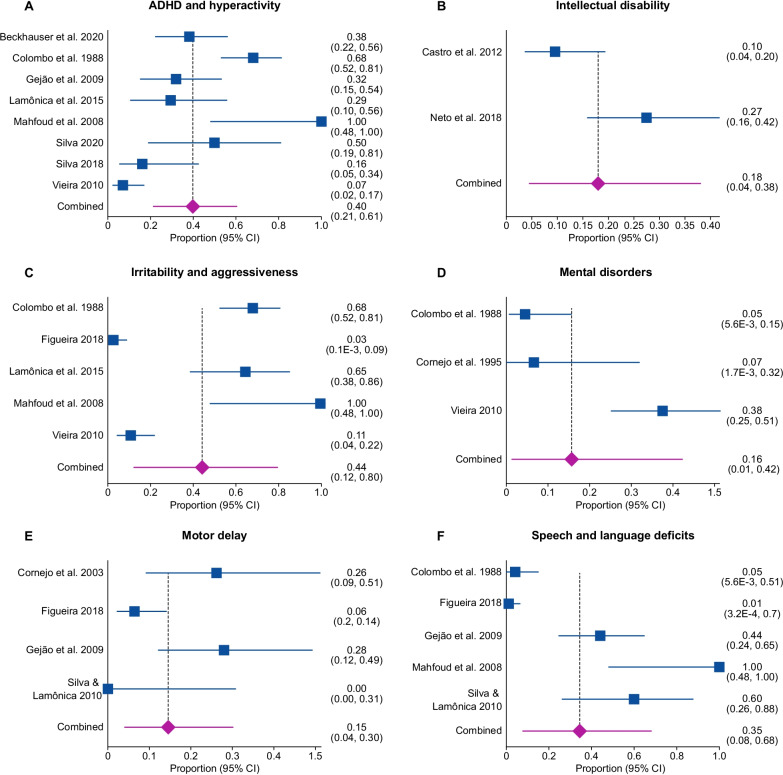
Proportional meta-analysis of neurological, neurocognitive, and neuropsychiatric impairments outcomes in early-diagnosed PKU patients. (**A**) ADHD and hyperactivity. (**B**) Intellectual disability. (**C**) Irritability and aggressiveness. (**D**) Mental disorders. (**E**) Motor delay**. (F**) Speech and language deficits

The pooled proportion of ADHD and hyperactivity was 40% [95% CI 0.21 to 0.61; I^2^ = 89.2%, *p* < 0.0001] from eight studies [29, 31, 39, 53, 59, 60, 78, 83, 98] with a total of 222 patients (Fig. 3A). There was significant statistical heterogeneity in the analyses.

##### Autism, intellectual disability, irritability and aggressiveness

A single study [49] evaluated early-diagnosed PKU patients who were reported to have autism. Out of 78 patients assessed, two were diagnosed with autistic behaviour.

The pooled proportion of intellectual disability was 18% [95% CI 0.04–0.38; I^2^ = 83.7%, *p* = 0.0133] from two studies [67, 77, 81] including a total of 114 patients (Fig. 3B). There was significant statistical heterogeneity in the analyses.

The pooled proportion of irritability and aggressiveness was 44% [95% CI 0.12–0.80; I^2^ = 96.2%, *p* < 0.0001] from five studies [22, 29, 39, 59, 60, 83] with a total of 200 patients (Fig. 3C). There was significant statistical heterogeneity in the analyses.

##### Mental disorders

The pooled proportion of mental disorder was 16% [95% CI 0.01–0.42; I^2^ = 89.7%, *p* < 0.0001] from three studies [29, 39, 42, 83] with a total of 115 patients (Fig. 3 D). A study [42] that reported slight retardation was also considered in this analysis. There was significant statistical heterogeneity in the analyses.

##### Motor delay

The pooled proportion of motor delay was 15% [95% CI 0.04–0.30; I^2^ = 74.5%, *p* = 0.0083] from four studies [22, 27, 41, 53, 98] with a total of 132 patients (Fig. 3E). There was significant statistical heterogeneity in the analyses. Any report of motor delay such as low motor development, neuromotor restriction [22], and deficit for gross motor area [27, 98] was considered.

##### Speech and language deficits

The pooled proportion of speech and language deficits was 35% [95% CI 0.08–0.68; I^2^ = 93.9%, *p* < 0.0001] from five studies [22, 27, 39, 53, 60, 98] with a total of 162 patients (Fig. 3F). There was significant statistical heterogeneity in the analyses. Additional reports of speech delay included speech deficits such as “only emits a word” [32], alterations in language [53], and mild to moderate speech disorder [27, 98] were reported.

#### Other comorbidities

##### Obesity and overweight

**Fig. 4:**
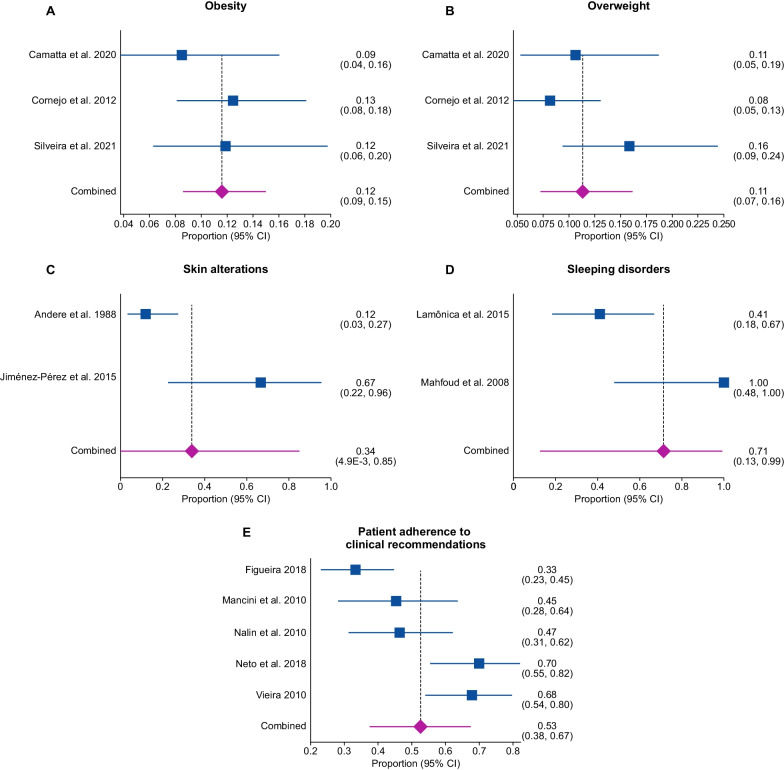
Proportional meta-analysis of other outcomes in early-diagnosed PKU patients. (**A**) Obesity. (**B**) Overweight. (**C**) Skin alterations. (**D**) Sleeping disorders. (**E**) Patient adherence to clinical recommendations

The pooled proportion of obesity was 12% [95% CI 0.09–0.15; I^2^ = 0%, *p* = 0.6129] from three studies [40, 71, 76, 80] with a total of 379 patients (Fig. 4, panel A). There was no significant statistical heterogeneity in the analyses.

The pooled proportion of overweight was 11% [95% CI 0.07–0.16; I^2^ = 47.2%, *p* = 0.1504] from three studies [40, 71, 76, 80] with a total of 379 patients (Fig. 4B). There was no significant statistical heterogeneity in the analyses.

##### Osteopenia

Only one study [43, 78] evaluating early-diagnosed PKU patients reported on osteopenia. Out of 31 patients, three were diagnosed with osteopenia.

##### Skin alterations

The pooled proportion of skin alterations was 34% [95% CI 4.9E-3 to 0.85; I^2^ = 85.7%, *p* = 0.0081] from two studies [55, 96] with a total of 40 patients (Fig. 4 C). Both included studies reporting lightening of the skin. There was significant statistical heterogeneity in the analyses.

##### Sleeping disorders

The pooled proportion of sleeping disorders was 71% [95% CI 0.13–0.99; I^2^ = 86.2%, *p* = 0.007] from two studies [59, 60] with a total of 22 patients (Fig. 4D). There was significant statistical heterogeneity in the analyses.

##### Patient adherence to clinical recommendations after treatment

The pooled proportion of patient adherence to clinical recommendation was 53% [95% CI 0.38 to 0.67; I^2^ = 83.7%, *p* < 0.0001] from five studies [22, 29, 62, 66, 67, 83] with a total of 260 patients (Fig. 4E). There was significant statistical heterogeneity in the analyses.

##### Socioeconomic impact

**Fig. 5:**
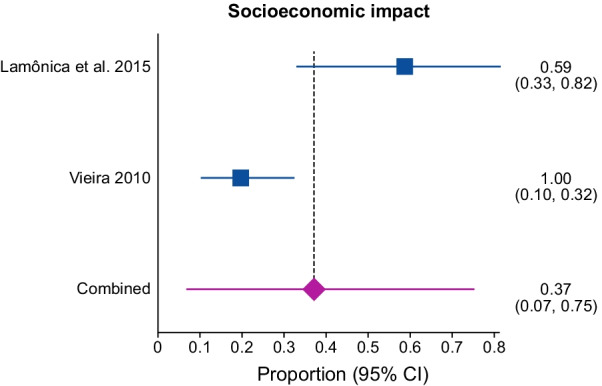
Proportional meta-analysis of economic outcomes in early-diagnosed PKU patients

The pooled proportion of socioeconomic impact was 37% [95% CI 0.07–0.75; I^2^ = 88.5%, *p* = 0.0032] from two studies [29, 59, 83] with a total of 73 patients (Fig. 5). There was significant statistical heterogeneity in the analyses. The included studies reported the following socioeconomic impact: poor school performance [18], and special education [29, 83].

##### Impact of phenylketonuria on caregiver health-related quality of life

The pooled proportion of impact of PKU on caregiver health-related quality of life (ie, did not acquire toilet training [32, 86]) was 42% [95% CI 0.09–0.80; I^2^ = 0%, *p* = 0.7519] from two studies [32, 86] with a total of five patients. There was no significant statistical heterogeneity in the analyses.

### Descriptive analysis of single studies reporting the outcomes of interest

Four studies [24, 58, 59, 61] reported executive function outcomes with 41% being classified as below average in the assessment of receptive vocabulary using the Peabody Image Vocabulary Test [59]. Malloy-Diniz et al. [61] reported that PKU children with high blood Phe levels (ie, mean Phe levels between 360 and 600 μmol/L) performed significantly worse than both the PKU children with low blood Phe levels and the control children on tasks that assess executive functioning. Morão et al. [24] found that the patients also showed a loss in the score of the Children Gambling Task. Lamônica et al. [58] reported that out of 10 patients, two of them presented outside the normality standards in the development scales. The skills were related to performance in motor, linguistic and cognitive activities. Furthermore, Poloni et al. [7] reported that most LATAM countries did not have low-protein foods, including Phe-free amino acid fortified, and no alternative treatments available. Also, they found that low purchasing power, limited/insufficient availability of low-protein foods, poor adherence, and lack of technical resources to manage the diet were major barriers to treatment. And last, Martins et al. [30] reported that half of the parents and caregivers who completed the survey had financial burden related to PKU management, some had to stop working to care for the PKU patient, and others had to hire a caregiver to assist the PKU patient. With regards to patient’s complaints, irritability was the most reported affected symtom accounting for 78% of the patients, followed by anxiety (67%), and lack of concentration (58%). Despite these findings, 70% of the patients have never undergone a cognitive and/or executive function assessment, and limitation on social activities, impact on professional life, and the effect on self-esteem were also listed as barriers to receive appropriate assessments.

## Discussion

### Main findings

PKU is a genetic inborn error in the metabolism of Phe. The pathogenic variants that cause PKU are present in high frequency in some LATAM countries such as Brazil and Chile [102].

Based on pooled data from 21 case series and cross-sectional studies [19, 22–24, 26, 29, 31–33, 39, 40, 42, 44, 52, 56, 59, 60, 67, 70, 71, 78, 80–82, 84–86, 88, 96–98] including 1224 patients, we found evidence demonstrating the impact of PKU on affected individuals in LATAM, with pooled proportions of burden ranging from 9% with osteopenia to 53% with speech and language deficits. Furthermore, only 53% of patients were adherent to clinical recommendations with 37% of patients experiencing socioeconomic impact of PKU. These are higher rates as compared to what we were expecting given that there is the ability to effectively diagnose and treat PKU.Query

### Strengths and limitations

Strengths of our review include a comprehensive search; assessment of eligibility, risk of bias and data abstraction independently and in duplicate; and an assessment of risk of bias that included a sensitivity analysis addressing homogeneity of study designs.

The primary limitation of our study is the highly heterogeneous nature of study samples in all studied clinical burden outcomes, except for the outcomes of obesity (Fig. 4, Panel A), osteopenia, and impact of PKU on caregiver health-related quality of life. Sources of this heterogeneity include both clinical and methodological diversities. The studies differed considerably in their mean age of patient selection, phenotype, modalities of implementation of the treatment (eg, newborn screening, access to treatment, lack of knowledgeable caregivers), and study designs (ie, case series and cross-sectional).

Furthermore, out of the 79 studies that met selection criteria, we were only able to include data in the meta-analysis from 21 of them (26.6%). The majority of the studies provided data on only one pre-specified outcome of interest, resulting in small sample sizes for many of the pooled analyses. In addition, there were studies that reported on late diagnosis patients and they were not included in the meta-analysis.

### Relation to prior research

One systematic review [103] identified in the literature corroborates our findings showing that even with dietary treatment, long-term physical growth (ie, body weight, height/recumbent length, and body mass index) are not attained in PKU. Another systematic review [104] showed that bone mineral density was lower in PKU patients compared with a control group. With regards to the latter outcome, four studies [105–109] reported a prevalence of osteopenia and osteoporosis ranging from 5 to 14%, which encompass our findings. Although we did not evaluate anthropometric variables in our review, we found a reasonable high prevalence of overweight individuals (11%) and of obesity (12%).

Furthermore, a frequent prevalence of being overweight was described in another systematic review [110] ranging from 7.8 to 32.6% in children and adolescents with PKU, which is also consistent with our findings (23%).

A very high prevalence of ADHD and hyperactivity (40%) and a moderate rate of intellectual disability (19%) were found in our review, which is consistent with others systematic reviews [111, 112] indicating that they are more common in both children and adults with PKU, despite being early diagnosed.

One study [113] conducted in the United States (US) showed that compared to the general population, PKU was associated with a significantly higher prevalence for intellectual disability, autism spectrum disorder, Tourette/tic disorders, eating disorders and behavior/conduct disorder in adult population. Of note, increased prevalence of these comorbidities persisted even when the sample was restricted to younger adults (aged 20–38 years), a subgroup with high probality of being diagnosed at birth and had the opportunity for continuos treatment throughout life. In parallel, a German study [114] not only corroborated that adults PKU patients suffered with neurospycholigical disease burden, but also revealed that this population presented additional comorbidities such as cardiometabolic risk factors. Also, these authors reported a higher intake of prescriptions for gastrointestinal agents, analgesics, antipyretics, statins, and antidepressants. Despite the methodological differences (both studies evaluated adult populations from a single country and were based on data retrieved from their respective healthcare systems), both studies are in line wth our findings that PKU potentially increases the neuropsychological comorbidities.

## Conclusions

LATAM PKU patients presented with a high prevalence of clinical complications, regardless of whether there is the possibility of residual confounding due to publication bias and the high heterogeneity in the analysis. Although it is widely accepted that PKU treatment is needed for life, the current approach in LATAM is primarily by using dietary management, which does not seem sufficient to avoid the disease burden outcomes investigated in this research. Furthermore, this review showed that there is a high degree of poor adherence to clinical recommendations. This study also highlights the need to address well-conducted burden of illness studies in PKU patients in LATAM to further elucidate the full spectrum of complications seen in this disease, to inform the healthcare providers taking care of these patients as well as the public health authorities on the ongoing and significant complications of this genetic disorder. [115–118]

## Supplementary Information

**Additional file 1: Table 1** Search strategy.

**Additional file 2: Table 2** Fifteen LATAM PKU included studies evaluating other outcomes than those pre-specified as patient-important or economic burden outcomes of interest.

**Additional file 3: Table 3** Reported pre-specified patient-important or economic burden outcomes on 12 LATAM PKU case reports studies.

**Additional file 4: Table 4** Risk of bias for cross-sectional studies.

**Additional file 5: Table 5** Risk of bias for case series studies.

**Additional file 6:** Data extraction, risk of bias assessment, subgroup and sensitivity analyses, heterogeneity assessment and publication bias.

## Data Availability

All data generated or analysed during this study are included in this published article and its Additional file 6: information files.

## Abbreviations

ADHD: Attention deficit hyperactivity disorder
ACMG: American College of Medical Genetics and Genomics guidelines
HPA: Hyperphenylalaninemia
LATAM: Latin American
PRISMA: Preferred reporting items for systematic review and meta-analysis
MOOSE: Meta-analysis of observational studies in epidemiology
PAH: Phenylalanine hydroxylase deficiency
PKU: Phenylketonuria
PROSPERO: International prospective register of systematic reviews
MeSH: Medical subject headings
MEDLINE: Medical literature analysis and retrieval system online
EMBASE: Excerpta medica database
CENTRAL: Cochrane central register of controlled trials
LILACS: Latin American and Caribbean Health Sciences Literature
SciELO: Scientific electronic library online
IBECS: Spanish bibliographic index of the health sciences
BINACIS: National bibliography in health sciences Argentina
MedCarib: Caribbean health sciences literature
CUMED: National Medical Sciences Information Center of Cuba
BBO: Brazilian bibliography of dentistry
ANVISA: National health surveillance agency
BDTD: Brazilian digital library of theses and dissertations
BMI: Body mass index
JBI: Joanna Briggs Institute
CI: Confidence interval
PROSPERO: (International Prospective Register of Systematic Reviews)
