# Supplementary material for: Burden of phenylketonuria in Latin American patients: a systematic review and meta-analysis of observational studies
Source: Orphanet J Rare Dis. 2022 Jul 30;17:302. doi: 10.1186/s13023-022-02450-2 (PMC9338521; doi:10.1186/s13023-022-02450-2)
Supplement: Supplementary file 1 — Additional file 1: Table 1 Search strategy. [file 13023_2022_2450_MOESM1_ESM.docx]

**Supplementary table 1.** Search strategy.

| ((phenylketonuria OR phenylketonurias OR PKU OR foelling disease OR folling disease OR mckusick 26160 OR Phenylketonuria II OR Dihydropteridine Reductase Deficiency OR Quinoid Dihydropteridine Reductase Deficiency OR DHPR Deficiency OR Atypical PKU OR HPABH4C OR Atypical Phenylketonuria OR Hyperphenylalaninemia, BH4-Deficient, C OR Due To DHPR Deficiency Tetrahydrobiopterin-Deficient Hyperphenylalaninemia OR Dihydropteridine Reductase Deficiency Disease OR Phenylketonuria Type 2 OR QDPR Deficiency OR Phenylketonuria I OR Folling Disease OR Folling's Disease OR Phenylalanine Hydroxylase Deficiency OR Classical Phenylketonuria OR PAH Deficiency OR Phenylalanine Hydroxylase Deficiency Disease OR Severe Phenylalanine Hydroxylase Deficiency Disease OR Oligophrenia Phenylpyruvica OR Hyperphenylalaninaemia OR Hyper-phenylalaninaemia OR Hyperphenylalaninaemias OR Hyper-phenylalaninaemias OR Non-Phenylketonuric Hyperphenylalaninemias OR Non-Phenylketonuric Hyperphenylalaninemia OR Non Phenylketonuric Hyperphenylalaninemia OR Hyperphenylalaninemia Caused by a Defect in Biopterin Metabolism OR Tetrahydrobiopterin Deficiency OR BH4 Deficiency OR hyperphenylalaninemia OR Phenylalanine OR Phe OR L-Isomer Phenylalanine OR L Isomer Phenylalanine OR L-Phenylalanine OR Endorphenyl OR Phenylalanine hydroxylase OR Phenylalanine 4-Hydroxylase OR Phenylalanine 4 Hydroxylase OR Phenylalanine 4-Monooxygenase OR Phenylalanine 4 Monooxygenase) AND (case-cohort study OR case-cohort studies OR case-control study OR case-control studies OR nested case-control OR cohort study OR cohort studies OR case series OR case report OR case reports OR observational study OR observational studies OR cross-sectional study OR cross-sectional studies OR cross sectional study OR cross sectional studies)) AND NOT (in vivo OR in vitro OR in vivo study OR in vitro study OR in vivo studies OR in vitro studies OR mutation study OR mutations study OR mutation studies OR mutations studies) |
| --- |
