# Supplementary material for: Burden of phenylketonuria in Latin American patients: a systematic review and meta-analysis of observational studies
Source: Orphanet J Rare Dis. 2022 Jul 30;17:302. doi: 10.1186/s13023-022-02450-2 (PMC9338521; doi:10.1186/s13023-022-02450-2)
Supplement: Supplementary file 2 — Additional file 2: Table 2 Fifteen LATAM PKU included studies evaluating other outcomes than those pre-specified as patient-important or economic burden outcomes of interest. [file 13023_2022_2450_MOESM2_ESM.doc]

**Supplementary table 2.** Fifteen LATAM PKU included studies evaluating **other outcomes than** those pre-specified as patient-important or economic burden outcomes of interest.

| Author, year | LATAM country | Scenario | # of participants | Age, Mean (SD), y | Female, % | Phenotype (%) | Early or late diagnosed** | Specify the type of treatment | Age (days) at start of treatment | Follow-up (months) | Outcomes evaluated other than those established in this review |
| --- | --- | --- | --- | --- | --- | --- | --- | --- | --- | --- | --- |
| Amorim et al., 2011 [19] | Brazil | Reference Service in Neonatal Screening in Bahia. | 111 | NR | 52.3 | Classic (56.8), mild (22.5) and HPA (19.8) | Early | NR for PKU, and a non restrictive diet for HPA. | 40 days for classic, 51 days mild, and 64 days HPA. | NA | Incidence; consanguinity; and income. |
| Azcoiti et al., 2020 [21] | Argentina | Niños Ricardo Gutiérrez Hospital | 27 | NR | 33.3 | Classic (22.2), moderate (29.6), mild (29.9) or PHPA (22.2) | NR | NR | NR | NA | Molecular diagnosis. |
| Cornejo et al., 2005 [39] | Chile | Neuropsychology Unit, INTA, University of Chile. | 29 | NR | 55 | NR | Early | Phenylalanine restricted diet. | NR | NA | Lipid composition of th diet. |
| Enacán et al., 2019 [46, 47] | Argentina | Sor Maria Ludovica Children’s hospital. | 420 | NR | 50.5^¥^ | Classic (25.2), moderate (27.2), mild (18.5), and HPA (29.1) | NR | NR | NR | NA | Genotype; and mutation spectrum prevalence. |
| Esteves et al., 1990 [49] | Brazil | APAE (Association of Parents and Friends of the Exceptional) and Neuropsychiatric Clinic of Alfenas -MG. | 15 | NR | 40 | NR | Late | NR | NA | NA | Incidence. |
| Fagioli et al., 2014 [50] | Brazil | Association of Exceptional Parents and Students (APAE), city of São Paulo. | 31 | NR | 45.16 | NR | Early | Diet and aminoacid formula diet. | Up to 180 days of life. | NA | Dietary habits. |
| Fisberg et al., 1999^£^ [53] | Brazil | Association of Parents and Friends of Handicapped Children (Associação de Pais e Amigos dos Excepcionais - APAE). | 42 | NR | NR | NR | NR | Phenylalanine restricted diet. | NR | NA | Nutritional status. |
| Kanufre et al., 2007 [58] | Brazil | Special Department of Genetics, Hospital das Clínicas, UFMG (SEG-HC-UFMG). | 70 | NR | 40 | NR | Early | Group 1: direct breast feeding and special formula without phenylalanine; and, group 2: commercial milk-based formula. | Group 1: 23; and Group 2: 24 | 12 | Effects of breastmilk. |
| Monteiro, 2006 [70] | Brazil | Regional Neonatal Screening Services in Brazil. | 1225 | NR | NR | NR | NR | NR | NR | NA | Identify the cause, symptoms, dietary treatment, national and international prevalence, and to the incidence in Brazil. |
| Oliveira, 2001 [74] | Brazil | Department of Pediatric Dentistry, Faculty of Dentistry, University of São Paulo (USP) in partnership with the Ecumenical Foundation for the Protection of the Exceptional (F.E.P.E.). | 40 | 9.3 | 50 | NR | NR | Phenylalanine restricted diet. | NR | NA | Oral health condition. |
| Ribeiro et al., 2012^£^ [82] | Brazil | Center for Research in Diagnostic Support (NUPAD), Faculty of Medicine, Federal University of Minas Gerais (UFMG). | 12 | 10.4 | 50 | Classic | Early | Phenylalanine restricted diet. | 34.6 | NA | Auditory pathway. |
| Russo-Estavillo et al., 2018 [83] | Mexico | Regional Hospital “Lázaro Cárdenas”, nº 46, Mexican Social Security Institute (IMSS) in Guadalajara, Jalisco, Mexico. | 17 | 3.55 | 35.3 | Classic (58.8), mild (11.8), moderate (5.9) and HPA (23.5) | Early | NR | Up to 2 days of life | NA | Genotypes, metabolic and clinical phenotypes. |
| Santos et al., 2011 [85] | Brazil | Special Department of Genetics Clinics Hospital UFMG (SEG-HC-UFMG). | 78 | NR | 43.58 | NR | Early | Group 1: direct breast feeding; Group 2: special low-phenylalanine formula. | Up to 40 days of life. | At least 30 days. | Energy, tyrosine and protein intakes of breastfed infants. |
| Starling, 2005^£^ [96] | Brazil | Phenylketonuria Outpatient Clinic of the Special Genetics Service of Hospital das Clínicas, UFMG (SEG-HC-UFMG). | 49 | 6.1 | 45 | NR | Early | Phenylalanine restricted diet. | NR | NR | Bone mass gain. |
| Stranieri & Takano, 2009 [98] | Brazil | Reference Service in Neonatal Screening for congenital hypothyroidism and phenylketonuria in the State of Mato Grosso. | 2 | 7.5 | NR | NR | Early | NR | 74 | NA | To evaluate the Reference Center for Neonatal Screening for congenital hypothyroidism and PKU. |

HPA: hyperphenylalaninemia; Phe: phenylalanine; PKU: phenylketonuria; LATAM: Latin America; NR: not reported; NA: not applicable; PHPA: hyperphenylalaninemia; SD: standard deviation.

#number.

*months.

**We considered that late diagnosed refers to children diagnosed between the ages of 3 months to 7 years (≥3 months - <7 years); untreated PKU refers to patients untreated by 7 years of age and over.

^¥^Genotype was carried out in 103 patients, therefore the percentage of female is based on 103 patients.

^£^Comparative cross-sectional study.
