# Supplementary material for: Burden of phenylketonuria in Latin American patients: a systematic review and meta-analysis of observational studies
Source: Orphanet J Rare Dis. 2022 Jul 30;17:302. doi: 10.1186/s13023-022-02450-2 (PMC9338521; doi:10.1186/s13023-022-02450-2)
Supplement: Supplementary file 3 — Additional file 3: Table 3 Reported pre-specified patient-important or economic burden outcomes on 12 LATAM PKU case reports studies. [file 13023_2022_2450_MOESM3_ESM.doc]

**Supplementary table 3.** Reported pre-specified patient-important or economic burden outcomes on 12 LATAM PKU case reports studies.

|  | **Individual level** | **Mortality** | **Physical** | **Psychosocial** | | **Others** | **Population level** | **Socioeconomic** | **Environmental** | **Health economic impact** |
| --- | --- | --- | --- | --- | --- | --- | --- | --- | --- | --- |
| **Blanco et al., 2012 [25]** |  | NA^£^ | | | | |  | NA^£^ | | |
| **De Lucca et al., 2017 [43]** |  |  | X^¢^ | X^¥^ | |  |  |  |  |  |
| **Escaf, 2003 [48]** |  |  |  | X^€^ | |  |  |  |  |  |
| **Figueiró-Filho 2004 [52]** |  |  |  |  | X* | |  |  |  |  |
| **Mariño & Zarzalejo, 2000 [66]** |  | NA^£^ | | | | |  | NA^£^ | | |
| **Menezes et al., 2019 [69]** |  |  | X^¢^ | X^€^ | |  |  | X ^ω^ |  |  |
| **Patricio & Maritza, 2018 [76]** |  | NA^£^ | | | | |  | NA^£^ | | |
| **Pereda-Torales et al., 2008 [77]** |  | NA^£^ | | | | |  | NA^£^ | | |
| **Rasner et al., 2014 [81]** |  |  | X^¢^ | X^€^ | |  |  |  |  |  |
| **Santos & Haack, 2013 [86]** |  | NA^£^ | | | | |  | NA^£^ | | |
| **Schmidt et al., 2016 [87]** |  | NA^£^ | | | | |  | NA^£^ | | |
| **Urbanes et al., 2006 [103]** |  |  | X^¢^ | X^$^ | |  |  | X^§^ |  |  |

^£^Case report study that did not evaluate any pre-defined burden outcomes.

*Maternal phenylketonuria.

^¢^Psychomotor retardation.

^¥^Severe mental retardation an autism.

^€^Irritability.

^ω^Delay in school performance.

^$^Aggressiveness and intellectual deterioration.

^§^Poor socialization, for which he was withdrawn from formal school.
