# Supplementary material for: Burden of phenylketonuria in Latin American patients: a systematic review and meta-analysis of observational studies
Source: Orphanet J Rare Dis. 2022 Jul 30;17:302. doi: 10.1186/s13023-022-02450-2 (PMC9338521; doi:10.1186/s13023-022-02450-2)
Supplement: Supplementary file 4 — Additional file 4: Table 4 Risk of bias for cross-sectional studies. [file 13023_2022_2450_MOESM4_ESM.doc]

**Supplementary table 4.** Risk of bias for cross-sectional studies.

| Author, year | Were the aims of the study clear? | Was the study design appropriate for the stated aims? | Was the sample size justified? | Was the target population clearly defined? | Was the sample frame taken from an appropriate population? | Were measures undertaken to address non-responders? | Were the risk factor and outcomes measured appropriate to the stated aims? | Were outcomes measured correctly using instruments that had been validated previously? | Is it clear what was used to determined statistical significance? | Were the demographic data adequately described? |
| --- | --- | --- | --- | --- | --- | --- | --- | --- | --- | --- |
| Andere et al., 1988^£^ [20] | Partially yes | Definitely yes | Definitely no | Definitely no | Unclear | Unclear | Partially yes | Unclear | Definitely no | Definitely no |
| Beckhauser et al., 2020 [22] | Definitely yes | Definitely yes | Definitely yes | Definitely yes | Definitely yes | Unclear | Partially yes | Definitely yes | Definitely yes | Partially yes |
| Brandalize & Czeresnia, 2004 [26,27] | Definitely yes | Definitely yes | Definitely yes | Definitely yes | Definitely yes | Unclear | Partially yes | Definitely yes | Definitely yes | Definitely yes |
| Camatta et al., 2020 [28,29] | Definitely yes | Definitely yes | Partially yes | Definitely yes | Definitely yes | Unclear | Definitely yes | Definitely yes | Definitely yes | Definitely yes |
| Pardo-Campos et al., 2021 [30-32] | Definitely yes | Definitely yes | Partially yes | Definitely yes | Definitely yes | Unclear | Definitely yes | Definitely yes | Definitely yes | Definitely yes |
| Castro et al., 2012 [33,34] | Definitely yes | Definitely yes | Definitely yes | Definitely yes | Definitely yes | Unclear | Partially yes | Definitely yes | Definitely yes | Partially yes |
| Chiesa et al., [36] | Definitely yes | Definitely yes | Partially yes | Definitely yes | Definitely yes | Unclear | Partially yes | Partially yes | Definitely no | Partially yes |
| Colombo et al., 1988 [37] | Definitely yes | Definitely yes | Definitely no | Definitely yes | Definitely yes | Unclear | Partially yes | Partially yes | Definitely no | Partially yes |
| da Silva et al., 2018 [42] | Definitely yes | Definitely yes | Definitely no | Definitely yes | Definitely yes | Unclear | Definitely yes | Definitely yes | Definitely yes | Definitely yes |
| Dutra, 2013^£^ [45] | Definitely yes | Definitely yes | Partially yes | Definitely yes | Definitely yes | Unclear | Partially yes | Definitely yes | Definitely yes | Partially yes |
| Gejão et al., 2009 [54] | Definitely yes | Definitely yes | Partially yes | Definitely yes | Definitely yes | Unclear | Partially yes | Definitely yes | Definitely yes | Partially yes |
| Keselman 2005 et al., [59]^£^ | Definitely yes | Definitely yes | Definitely no | Definitely yes | Unclear | Unclear | Definitely yes | Definitely yes | Unclear | Partially yes |
| Kanufre et al., 2015 [57] | Definitely yes | Definitely yes | Partially yes | Definitely yes | Definitely yes | Unclear | Partially yes | Definitely yes | Definitely yes | Partially yes |
| Lamônica et al., 2015 [62] | Definitely yes | Definitely yes | Partially yes | Definitely yes | Definitely yes | Unclear | Partially yes | Definitely yes | Definitely yes | Definitely yes |
| Malloy-Diniz et al., 2004^£^ [64] | Definitely yes | Definitely yes | Partially yes | Definitely yes | Definitely yes | Unclear | Definitely yes | Definitely yes | Definitely yes | Definitely yes |
| Mancini et al., 2010^£^ [65] | Definitely yes | Definitely yes | Partially yes | Definitely yes | Definitely yes | Unclear | Definitely yes | Definitely yes | Definitely yes | Partially yes |
| Martins et al., 2021 [67] | Definitely yes | Definitely yes | Partially yes | Definitely yes | Definitely yes | Unclear | Definitely yes | Definitely yes | Unclear | Definitely yes |
| Mendes, 2006 [69] | Definitely yes | Definitely yes | Partially yes | Definitely yes | Definitely yes | Unclear | Partially yes | Definitely yes | Definitely yes | Partially yes |
| Morão, 2017 [71] | Definitely yes | Definitely yes | Partially yes | Definitely yes | Partially yes | Unclear | Unclear | Definitely yes | Unclear | Unclear |
| Nalin et al., 2010 [72] | Definitely yes | Definitely yes | Partially yes | Definitely yes | Definitely yes | Unclear | Partially yes | Unclear | Definitely yes | Definitely yes |
| Vieira-Neto et al., 2018^£^ [73] | Definitely yes | Definitely yes | Definitely yes | Definitely yes | Definitely yes | Unclear | Definitely yes | Definitely yes | Definitely yes | Definitely yes |
| Paneque et al., 2013 [75] | Definitely yes | Definitely yes | Partially yes | Definitely yes | Definitely yes | Unclear | Definitely yes | Definitely yes | Unclear | Partially yes |
| Peredo et al., 2010 [78] | Unclear | Unclear | Partially yes | Partially yes | Definitely yes | Unclear | Definitely yes | Definitely yes | Definitely yes | Partially yes |
| Pérsico et al., 2019 [79] | Definitely yes | Definitely yes | Definitely yes | Definitely yes | Definitely yes | Unclear | Definitely yes | Definitely yes | Definitely yes | Partially yes |
| Poloni et al., 2021 [7] | Definitely yes | Definitely yes | Partially yes | Partially yes | Definitely yes | Unclear | Definitely yes | Definitely yes | Unclear | Definitely no |
| Sena, 2018 [88] | Definitely yes | Definitely yes | Partially yes | Definitely yes | Definitely yes | Unclear | Partially yes | Definitely yes | Definitely yes | Partially yes |
| Silva & Lamônica, 2010^£^ [92] | Definitely yes | Definitely yes | Partially yes | Definitely yes | Definitely yes | Unclear | Partially yes | Definitely yes | Definitely yes | Definitely yes |
| Silva, 2016 [93] | Definitely yes | Definitely yes | Partially yes | Definitely yes | Definitely yes | Unclear | Partially yes | Partially yes | Definitely yes | Definitely yes |
| Silva, 2018 [89] | Definitely yes | Definitely yes | Definitely yes | Definitely yes | Definitely yes | Unclear | Partially yes | Partially yes | Definitely yes | Definitely yes |
| Silveira et al., 2021 [95] | Definitely yes | Definitely yes | Partially yes | Definitely yes | Definitely yes | Unclear | Definitely yes | Definitely yes | Definitely yes | Definitely yes |
| Teruya, 2019 [100,101] | Definitely yes | Definitely yes | Definitely yes | Definitely yes | Definitely yes | Unclear | Partially yes | Definitely yes | Definitely yes | Definitely yes |
| Tonon et al., 2019^£^ [102] | Definitely yes | Definitely yes | Definitely yes | Definitely yes | Definitely yes | Unclear | Partially yes | Definitely yes | Definitely yes | Definitely yes |
| Vieira, 2010 [105,106] | Definitely yes | Definitely yes | Partially yes | Definitely yes | Definitely yes | Unclear | Definitely yes | Partially yes | Definitely yes | Definitely yes |

Definitely yes = low risk of bias; partially yes = probably low risk of bias; partially no = probably high risk of bias; definitely no = high risk of bias; unclear = not enought information for a judgment.

^£^Comparative cross-sectional study.
