# Supplementary material for: Burden of phenylketonuria in Latin American patients: a systematic review and meta-analysis of observational studies
Source: Orphanet J Rare Dis. 2022 Jul 30;17:302. doi: 10.1186/s13023-022-02450-2 (PMC9338521; doi:10.1186/s13023-022-02450-2)
Supplement: Supplementary file 5 — Additional file 5: Table 5 Risk of bias for case series studies. [file 13023_2022_2450_MOESM5_ESM.docx]

**Supplementary table 5.** Risk of bias for case series studies.

| Author, year | Were patient’s demographic characteristics clearly described? | Was the current clinical condition (PKU) of the patient on presentation clearly described? | Were diagnostic tests or assessment methods and the results clearly described? | Was the intervention(s) or treatment procedure(s) clearly described? | Does the case report provide takeaway lessons? |
| --- | --- | --- | --- | --- | --- |
| Benítez et al., 2001 [23] | Partially yes | Partially yes | Definitely no | Definitely no | Definitely no |
| Bernal, 2017 [24] | Partially yes | Definitely yes | Definitely yes | Partially yes | Partially yes |
| Cornejo et al., 1995 [41] | Partially yes | Definitely yes | Definitely yes | Definitely yes | Partially yes |
| Cornejo et al., 2003 [40] | Partially yes | Definitely yes | Definitely yes | Definitely yes | Definitely yes |
| Cornejo et al., 2012 [38] | Definitely yes | Definitely yes | Definitely yes | Definitely yes | Definitely yes |
| Figueira, 2018 [51] | Definitely yes | Definitely yes | Partially yes | Partially yes | Partially yes |
| Gelvez et al., 2016 [55] | Definitely yes | Definitely yes | Definitely yes | Definitely yes | Partially yes |
| Jiménez-Péres et al., 2015 [56] | Definitely yes | Definitely yes | Definitely yes | Unclear | Partially yes |
| Lamônica et al., 2012 [61] | Definitely yes | Definitely yes | Definitely yes | Definitely no | Partially yes |
| Mahfoud et al., 2008 [63] | Partially yes | Definitely yes | Partially yes | Partially yes | Definitely yes |
| Martins, 2007 [67] | Partially yes | Definitely yes | Definitely yes | Definitely yes | Definitely yes |
| Queiroz & Pondé, 2015 [80] | Partially yes | Definitely yes | Partially yes | Definitely yes | Definitely yes |
| Sánchez-Peña et al., 2008 [84] | Definitely yes | Definitely yes | Definitely yes | Definitely yes | Definitely yes |
| Silva et al., 2016 [94] | Definitely yes | Definitely yes | Definitely yes | Partially yes | Partially yes |
| Steiner et al., 2007 [97] | Partially yes | Definitely yes | Definitely yes | Definitely yes | Definitely no |
| Tanaka et al., 2018 [99] | Partially yes | Definitely yes | Definitely yes | Definitely yes | Definitely yes |
| Valle et al., 2019 [104] | Partially yes | Partially yes | Partially yes | Definitely yes | Partially yes |

Definitely yes = low risk of bias; partially yes = probably low risk of bias; partially no = probably high risk of bias; definitely no = high risk of bias; unclear = not enought information for a judgment.
