# Supplementary material for: Burden of phenylketonuria in Latin American patients: a systematic review and meta-analysis of observational studies
Source: Orphanet J Rare Dis. 2022 Jul 30;17:302. doi: 10.1186/s13023-022-02450-2 (PMC9338521; doi:10.1186/s13023-022-02450-2)
Supplement: Supplementary file 6 — Additional file 6: Data extraction, risk of bias assessment, subgroup and sensitivity analyses, heterogeneity assessment and publication bias. [file 13023_2022_2450_MOESM6_ESM.docx]

**Data extraction**

Reviewers independently extracted the following data using a pre-standardized data extraction form: (a) first author and year of publication; (b) country; (c) study design; (d) scenario; (e) age and gender; (f) diagnoses (classic or mild PKU, HPA); however defined per the authors of the included studies; (g) eligibility criteria; (h) early diagnosis and early start of treatment (we considered that late diagnosed refers to children diagnosed between the ages of 3 months to 7 years (≥3 months to <7 years); untreated PKU refers to patients untreated by 7 years of age and over [3]); and (i) patient-important and economic outcomes, if available. We avoided double-counting of patients where there were multiple publications of the same population. If there was more than one published report of the same group of patients, the articles were analyzed to verify whether or not they reported different outcomes. If they presented the same outcomes, data was extracted from the most recent article.

**Risk of bias assessment**

We evaluated risk of bias assessment only for those studies that reported patient-important outcomes (n = 30 cross-sectional studies; n = 17 case series; no cohort or case-control studies assessed these outcomes).

For cross-sectional studies, we assessed risk of bias with the AXIS tool [10], though we excluded some domains not applicable to our review. For case series, we used the Joanna Briggs Institute (JBI) critical appraisal checklist [11]. Response options for both tools were modified to “definitely yes” (low risk of bias), “partially yes” (not all information needed available), “unclear” (no information to judge), and “definitely no” (high risk of bias), as we believe that this categorization is more reliable.

For cohort and case-control studies, we planned to assess risk of bias with a modified version of the Ottawa-Newcastle instrument [12] that includes confidence in assessment of exposure and outcome; however, there was no included study classified as either a cohort or case-control study assessing patient-important outcomes in order we could assess their risk of bias.

**Subgroup and sensitivity analyses**

There were several protocol stated pre-planned subgroup analyses provided there was a minimum of two studies in each category: (1) start of treatment (early diagnosed was considered up to three months of life, inclusively; (2) patients maintaining blood Phe in targeted range versus those not able to do so; (3) PKU impact evaluated by Patient-Reported Outcomes (PROs) and validated clinical tools (eg, questionnairies, scales) versus health care and parents/caregivers perception; (4) children versus adults; (5) countries with newborn screening available versus countries without; and (6) per LATAM countries (eg, Brazil versus Argentina). However due to insufficient number of studies, no subgroup analysis could be conducted. Further pre-planned sensitivity analysis to explore causes of heterogeneity of the results, separating studies according to study designs (eg, cross-sectional versus case series) were also not conducted due to insufficient number of studies to allow for these assessments.

**Heterogeneity assessment and publication bias**

We investigated heterogeneity using the chi-square test and the I^2^ statistic [16]. An I^2^ value of 0–40%, 30–60%, 50–90% or 75–100% was interpreted as not important, moderate, substantial, or considerable heterogeneity, respectively, and significance will be assumed when I^2^ was ≥50% with a P < 0.1.

There was an insufficient number of studies (at least 10 or more) to allow for assessment of publication bias through visual inspection of funnel plots. Review Manager (RevMan) provided the software for all analyses (version 5.3; Nordic Cochrane Centre, Cochrane) [17].
